# Caregiving experiences and support challenges of older caregivers from the perspective of the digital divide: a qualitative study

**DOI:** 10.3389/fpubh.2026.1797984

**Published:** 2026-08-04

**Authors:** Qi Liu, Lisha Xia, Chun Wu, Weina Dang, Juelian Li, Yali Liu, Chao Shang, Yu Deng, Jinxia Zhang

**Affiliations:** General Hospital of Southern Theater Command of PLA, Guangzhou, China

**Keywords:** digital divide, digital health, geriatric care, older caregivers, qualitative research, social support

## Abstract

**Purpose:**

This study aims to explore the caregiving experiences and support challenges faced by older caregivers when using digital health technologies. By examining these experiences through the lens of the digital divide, this research seeks to provide empirical evidence to support the development of inclusive and age-friendly digital health services.

**Methods:**

This study employed a qualitative descriptive design. Using purposive sampling, 16 older caregivers of hospitalized older patients were recruited from the cardiovascular department of a tertiary hospital in Guangzhou. Semi-structured interviews were conducted between July 6 and August 25, 2025, and data were analyzed using directed content analysis.

**Results:**

The experiences and dilemmas of older caregivers interacting with digital health technologies were categorized into three core themes and six sub-themes: (1) Access-level barriers (Mismatch between age-related decline and technology design; Lack of intergenerational support); (2) Operational challenges (Cognitive overload from limited digital literacy; Concerns regarding technology efficacy and safety); and (3) Gaps in perception and trust (Reliance on traditional caregiving methods; Insufficient professional guidance and help-seeking channels).

**Conclusion:**

This study shows that the digital divide faced by older caregivers extends beyond mere access or skills. It is also shaped by age-related decline, complex technology design, limited trust, and insufficient support. Cumulatively, these factors drive digital disability, compromising caregivers’ ability to navigate digital health services effectively in real-world caregiving scenarios. Addressing these challenges requires age-friendly design, targeted digital literacy training, and a multi-sectoral support network involving families, communities, healthcare institutions, and policymakers. These efforts are essential to ensure equitable access to digital health services and foster healthy aging.

## 1 Introduction

According to the World Health Organization, the global population aged 60 years and older is expected to reach 2.1 billion by 2050 (1). Driven by this demographic shift, family caregiving structures are evolving, with older adults increasingly providing care for spouses, relatives, or peers of a similar age (2, 3). In developed countries, approximately 25% of family caregivers are over the age of 65 (2), while in China, the proportion of older care has exceeded 40% (3). In this study, older caregivers refer to individuals aged 60 years or older who provide primary daily care for their older family members. Given their own age-related physical and psychological vulnerabilities, these caregivers often shoulder a substantial burden. This burden not only compromises their own physical and mental health, but negatively impacts the health outcomes of care recipients and the effectiveness of nursing interventions (4). Driven by the rapid proliferation of digital health technologies, digital health services and wearable devices are increasingly being integrated into home care settings, with adoption rates among older adults rising steadily (5). However, mere access to these technologies does not inherently translate into effective utilization. Many older caregivers encounter substantial barriers when attempting to incorporate digital health technologies into their daily caregiving routines. These challenges are often compounded by complex user interfaces, limited literacy, age-related physical and cognitive declines, and an absence of timely support. In China, policies have increasingly encouraged community-based care for older adults. At the same time, primary healthcare institutions often lack sufficient resources to provide continuous in-home nursing guidance, digital health support, and psychological assistance for older caregivers (6). Consequently, these caregivers find themselves confronting a dual burden: traditional caregiving pressure and new digital demands. When digital health services become part of routine care, difficulties in using these technologies may actually increase caregiving stress and health risks rather than reducing them (7).

As healthcare becomes increasingly digitized, DDT has widely adopted across disciplines such as public health, health informatics, and nursing. This theoretical framework specifically explores how technological exclusion exacerbates health inequities and impacts the quality of care (8). Classically, DDT is conceptualized through three sequential dimensions: (1) the access divide, which addresses inequities in physical access to digital health technologies and internet infrastructure; (2) the usage divide, which focuses on disparities in the ability to effectively use these technologies, including digital literacy, skills, and the frequency and purpose of their use; and (3) the knowledge divide, which pertains to inequalities in the ability to find, understand, evaluate, and apply digital health information, ultimately influencing health decision-making and care outcomes (9).

Existing studies on the digital divide among older adults have primarily focused on general internet or social media use, or have relied on quantitative surveys to identify influencing factors. Less attention has been paid to how older caregivers use digital health technologies in high-pressure caregiving situations, such as medication management, appointment scheduling, symptom monitoring, and emergency decision-making (8). Furthermore, previous research has often treated older adults as a broad and relatively homogeneous group of technology users (10). This approach may overlook the specific vulnerabilities of older caregivers, who must manage their own age-related decline while caring for another older adults. Moreover, much of the existing evidence comes from cross-sectional studies (11). While useful for identifying associated factors, these studies provide limited insight into how digital barriers are experienced in daily caregiving. In reality, there is considerable variation in the digital adaptability of older caregivers (7).

Therefore, this study applies the DDT as the core analytical framework to specifically examine older caregivers. Using a qualitative approach, this study explores the practical and support challenges faced by this population across the access, usage, and knowledge dimensions of the digital divide. The findings aim to provide empirical evidence for developing an inclusive, age-friendly digital health support system.

## 2 Methods

### 2.1 Participants and sampling

This qualitative study conducted individual semi-structured interviews with older caregivers of patients hospitalized in the cardiovascular department of a tertiary hospital in Guangzhou. Purposive sampling was used to recruit participants with varying educational backgrounds, caregiving durations, and smart device proficiency to ensure a diversity of perspectives (Table 1). Because cardiovascular disease requires long-term care and close monitoring, caregivers in this setting often rely heavily on digital health technologies. Focusing on this single-center, single-disease population enabled a more in-depth exploration of the digital divide within a specific, high-demand caregiving context. The inclusion criteria were as follows: (1) both the caregiver and the hospitalized patient were aged ≥60 years; (2) were the primary family caregiver, providing ≥4 h of daily care; (3) have at least 3 months of caregiving experience; (4) have used at least one digital health technologies for caregiving tasks within the past month; (5) have no cognitive impairment and can communicate effectively in Mandarin; and (6) they gave voluntary informed consent. Exclusion criteria included: (1) severe cognitive impairment; (2) acute or terminal illness; (3) severe hearing or visual impairment not correctable by assistive devices; and (4) participation in a similar study within the past 3 months. The final sample size of 16 participants was determined by data saturation (the point at which no new themes emerged).

**Table 1:** Participant characteristics.

ID	Gender	Age (years)	Education level	Relationship to patient	Daily care hours (h)	Number of chronic diseases	Smart device experience	Domicile
P1	Female	68	Primary School	Spouse	16	2	None	Rural
P2	Male	65	Junior High	Spouse	15	1	Minimal	Town/City
P3	Female	72	Illiterate	Sibling	8	3	None	Town/City
P4	Female	70	Primary School	Spouse	17	2	None	Rural
P5	Male	67	Junior High	Spouse	14	2	Minimal	Town/City
P6	Female	75	Primary School	Spouse	10	4	None	Town/City
P7	Female	63	Senior High	Children	15	1	Moderate	Rural
P8	Male	69	Junior High	Sibling	6	3	Minimal	Rural
P9	Female	66	Primary School	Spouse	11	2	None	Town/City
P10	Female	71	Illiterate	Spouse	9	3	None	Town/City
P11	Male	64	College	Spouse	14	1	Moderate	Town/City
P12	Female	73	Primary School	Spouse	18	3	None	Town/City
P13	Male	68	Junior High	Spouse	15	2	Minimal	Rural
P14	Female	74	Primary School	Spouse	17	3	None	Town/City
P15	Female	62	Senior High	Spouse	16	1	Moderate	Rural
P16	Male	70	Junior High	Spouse	15	2	Moderate	Rural

### 2.2 Ethics approval and considerations

The study protocol was approved by the Ethics Committee of the General Hospital of the Southern Theater Command of the PLA (Approval No.: NZLLKZ2025064; July 17, 2025) and registered with the Chinese Clinical Trial Registry (ChiCTR2500110918). All research procedures were conducted in strict accordance with the ethical principles of the Declaration of Helsinki. Written informed consent was obtained from all participants prior to their inclusion in the study.

### 2.3 Data collection

The interview guide was initially developed based on existing literature on DDT and subsequently reviewed by three experts in geriatric nursing and qualitative research. Prior to formal data collection, the guide was pilot-tested with two older caregivers; based on their feedback, the wording was simplified to ensure clarity and accessibility for an older population. All interviews were conducted by the lead author (Q.L.), a researcher experienced in qualitative interviewing techniques. The average duration of the interviews was 42 min (range: 30–60 min). Following informed consent, all interviews were audio-recorded and transcribed verbatim. To elicit in-depth descriptive narratives, the semi-structured interview guide focused on three primary dimensions: (1) Access divide: What do you find helpful about using these technologies for caregiving? What operational difficulties do you encounter? How do you resolve them, and what other concerns do you have? (2) Usage divide: Which medical applications or mini-programs do you typically use, and for what specific purposes? How would you evaluate their usability and effectiveness in supporting your daily caregiving tasks? What additional assistance or guidance do you expect from healthcare professionals? (3) Knowledge divide: How do you interpret and utilize digital health information? How do digital care models compare to traditional care methods, and which do you prefer?

### 2.4 Data analysis

Data were analyzed using a directed content analysis approach, facilitated by NVivo 14.0 software. Two researchers independently reviewed the transcripts and conducted the initial coding. Using the DDT as a deductive guide, the access, usage, and knowledge divides served as the foundational coding framework. Codes sharing similar meanings were then synthesized into broader categories to develop overarching themes and sub-themes. Any coding discrepancies were resolved through discussion to reach a consensus, with a third qualitative expert consulted when necessary.

## 3 Results

Participants articulated several critical barriers and adaptive challenges when navigating digital healthcare tasks within a geriatric caregiving context. Compounding practical barriers—such as complex user interfaces, inadequate technical support, and cognitive overload—significantly hindered their ability to perform caregiving tasks effectively. The hierarchical coding framework, detailing the progression from initial codes to overarching themes and sub-themes, is presented in Table 2.

**Table 2:** Subthemes and themes identified from coding process.

Sub-themes	Themes
Mismatch between age-related decline and technology design	Access-level barriers
Lack of intergenerational support	
Cognitive overload from limited digital literacy	Operational challenges
Concerns regarding technology efficacy and safety	
Reliance on traditional caregiving methods	Gaps in perception and trust
Insufficient professional guidance and help-seeking channels	

### 3.1 Access-level barriers

#### 3.1.1 Mismatch between age-related decline and technology design

Compounded by natural physiological aging, older caregivers frequently struggled with visual recognition and touchscreen navigation when interacting with smart health devices. Visual impairments, for instance, severely compromised usability. Participant P1 stated, “I have presbyopia and cannot read the small text on the phone at all. Even with reading glasses, the screen glare still makes it impossible to see clearly.” Participant P2 mentioned, “My fingers are thick and a bit shaky, so I always tap the wrong buttons. Sometimes I accidentally open other pages and cannot find my way back.” Participant P14 also reported, “I do not know how to use it, and I do not want to. You teach me, but I cannot remember.” Collectively, these fundamental operational hurdles emerged as the primary barriers to digital health adoption among older caregivers.

#### 3.1.2 Lack of intergenerational support

Family support is important for older caregivers attempting to navigate digital health technologies, younger relatives were frequently too preoccupied to offer sustained guidance. As P15 noted: “Only my son has a smartphone, and he’s usually not home.” P8 explained: “The kids are busy with work and do not have time to teach us again and again.” Consequently, when family support could not translate into practical, hands-on assistance, older caregivers frequently developed feelings of frustration, leading to increased hesitancy and resistance toward digital health adoption.

### 3.2 Operational challenges

#### 3.2.1 Cognitive overload from limited digital literacy

Older caregivers often had only a fragmented understanding of health management. This conceptual gap hindered their ability to comprehend and execute clinical guidance delivered via digital health technologies. The perceived complexity of multi-step procedures often led to immediate frustration. For instance, P9 noted that “scanning QR codes and downloading is too much of a hassle,” while P8 preferred direct, simplified instructions over digital interaction: “It’s too much trouble. Just tell me simply what I need to do at home.” This fundamental confusion was echoed by P4, who asked bluntly, “What do I need to do?” These multi-layered tasks imposed a substantial cognitive load that exceeded their digital proficiency. This sense of alienation from rapid technological shifts was further articulated by P7: “My mom can barely use a smartphone, and I’m at this age too. I cannot keep up with these modern trends.”

#### 3.2.2 Concerns regarding technology efficacy and safety

Many older caregivers maintained a conservative attitude toward technology. P7: “Machines are unreliable. What if a system error delays my treatment?” P11 questioned the system’s practical efficacy: “If I use this software to record my blood pressure and heart rate, can the doctor actually see it?” Furthermore, anxieties surrounding data privacy and potential financial exploitation compounded this reluctance. P13 voiced specific fears regarding surveillance: “I heard about location tracking; I’m afraid of getting scammed.”

### 3.3 Gaps in perception and trust

#### 3.3.1 Reliance on traditional caregiving methods

Extensive caregiving experience has equipped many older caregivers with deeply ingrained routines and a steadfast reliance on traditional methods. As P15 noted, “He has presbyopia and does not use a phone; he cannot use these things well when I’m not home.” Dismissing the need for technological oversight, P10 asserted, “I do not want to use it; I can take better care of him myself.” P6 emphasized, “I’ve taken care of him for over a decade; I can tell if he’s comfortable just by looking.”

#### 3.3.2 Insufficient professional guidance and help-seeking channels

Older caregivers articulated a pressing demand for standardized training and dedicated professional support. This lack of cross-institutional integration was highlighted by P10, who pointed out the redundancy in platform requirements: “The hospital told me to use an app when I was discharged, and the community asked me to use theirs. It would be better if they were unified.” P12 noted: “There are so many of these apps; I do not know which one is best.”

## 4 Discussion

### 4.1 Digital disability as a cumulative consequence of the digital divide

This qualitative study illuminates the complex barriers older caregivers encounter when navigating digital health services. Our findings reveal that these challenges extend far beyond the mere absence of hardware or internet connectivity. Rather, they expose a multidimensional deficit in physical, cognitive, and social resources requisite for effective digital engagement. While the primary data were gathered in a clinical setting, the implications resonate profoundly within home-based care paradigms, particularly for chronic conditions like cardiovascular diseases that necessitate vigilant, long-term management (12). As critical caregiving protocols increasingly migrate to digital health technologies, these technological frictions extend beyond the hospital setting, ultimately compromising the continuity and efficacy of daily care at home.

We observed that digital barriers do not occur as isolated incidents but can accumulate over time. Repeated technological friction, together with insufficient support, gradually undermines caregivers’ skills and confidence. This results in what we term digital disability, a chronic difficulty in using digital health technologies effectively that is shaped by age-related decline, poor system design, and weak social networks (13). While classic digital divide theory emphasizes access, our findings show that the ability to use digital health technologies safely, independently, and consistently is far more critical. When caregivers cannot easily book appointments or check test results, technology becomes an added burden rather than a source of support. Although direct clinical causality cannot be established, participants’ narratives suggest that these digital failures may increase caregiving stress and compromise perceived patient safety. Consistent with previous research (14), our study identifies a risk pathway through which digital disability hinders practical care delivery and diminishes trust in geriatric home-based care.

### 4.2 Structural exclusion and digital dilemmas among older caregivers

Caregivers’ difficulties arise when individual limitations intersect with rigid digital health technologies (10). Structural exclusion is most clearly reflected in user interfaces that fail to accommodate older adults’ needs, including tiny fonts, confusing layouts, excessive verification steps, and dense medical jargon. Such design features undermine usability and alienate older adults, leaving them feeling as though modern healthcare was not built with them in mind. Consistent with evidence from high-income countries and telemedicine research (15–17), simply providing older adults with access to technology is unlikely to be effective if digital systems are not designed to accommodate their specific needs.

Furthermore, while digital health research in high-income contexts increasingly focuses on artificial intelligence (18), our participants continued to struggle with more basic barriers, including unreliable assistance and limited digital skills. This contrast underscores the context-dependent nature of the digital divide. In rural and under-resourced areas, exclusion may be even more severe because infrastructure costs remain high (19). Digital health policies should therefore prioritize accessibility, usability, and assisted use rather than technological innovation for its own sake. In practice, this requires developers to comply with age-friendly design standards and policymakers to maintain non-digital and in-person service options so that vulnerable groups are not left behind during digital transitions.

### 4.3 Fragmented social support and the call for a multi-sectoral collaborative network

The fragmented social support identified in this study exacerbates the risk that older caregivers will be left behind in digital health transitions. Consistent with previous literature (20, 21), willingness to adopt digital health technologies is strongly shaped by living arrangements and psychological well-being. In the Chinese cultural context, the tradition of xiao positions adult children as key sources of care, problem-solving, and practical support (22). However, rapid urbanization, population mobility, and declining family size have weakened these traditional safety nets. Consequently, the absence of intergenerational assistance is not merely a technical barrier; it is also experienced as an emotional loss and an unmet familial expectation, deepening caregivers’ sense of helplessness when navigating unfamiliar technologies (23).

To handle massive patient volumes, major hospitals in China have rapidly shifted many essential services onto platforms such as WeChat. When older caregivers were required to use complex digital systems, they often struggled with online registration, administrative procedures, and medical instructions. If their children were away or at work, they had few people to turn to for help. This left them to manage feelings of stress and inadequacy alone (24). Going forward, digital health technologies help desks for older adults could be established, geriatric digital disability could be incorporated into national aging policies, and long-term care insurance could be strengthened. These measures may help reduce digital exclusion and promote more equitable access to healthcare.

## 5 Strengths and limitations

This study has two main strengths. First, it provides an in-depth qualitative understanding of digital barriers among older caregivers. By focusing on their lived experiences, the study shows how digital health technologies are used, avoided, or misunderstood in everyday caregiving contexts. This perspective adds contextual depth to previous studies that have mainly examined the digital divide in terms of access, device ownership, or general digital literacy. Second, this study focuses on older caregivers of patients with cardiovascular disease, a group that has received limited attention in digital health research. Cardiovascular care often requires long-term monitoring, medication management, appointment scheduling, and timely responses to symptoms. These care tasks are increasingly mediated through digital health technologies. Therefore, this study provides empirical insights into how physical decline, cognitive burden, limited trust, and fragmented support shape caregivers’ use of digital health services. It also helps explain how the digital divide may become a form of digital disability in high-pressure caregiving contexts.

This study also has several limitations. First, the transferability of the findings is limited. Participants were recruited from a single tertiary hospital, and the sample size was small. As a result, the findings may not fully reflect the experiences of older caregivers in other regions, hospital levels, or community-based care settings. Future studies should include participants from different geographic areas, healthcare institutions, and community contexts to determine whether similar digital barriers exist across diverse care environments. Second, the hospital-based setting may have influenced participants’ experiences. Caregivers interviewed in the hospital setting were still within a professional care environment and could seek help from healthcare workers when needed. Their experiences may differ from those of caregivers providing post-discharge care at home. In home settings, older caregivers may rely more heavily on digital health technologies and may experience greater anxiety when support is unavailable. Future research should follow caregivers across the transition from hospital to home care to better understand how digital barriers change over time and across care settings. Third, this study focused only on cardiovascular caregiving. This focus helped capture digital barriers related to monitoring, medication use, emergency response, and continuity of care. However, the findings may not fully apply to other chronic conditions. For example, caregivers of patients with dementia, cancer, or stroke may face different digital needs and caregiving pressures. Future studies should compare the forms and consequences of digital disability across different disease groups. Finally, this was a qualitative study and did not test any intervention. As a result, the effectiveness of possible strategies, such as digital skills training, simplified hospital platforms, or community-based support services, remains unknown. Future research should design, implement, and evaluate targeted interventions that combine age-friendly technology design, practical training, and timely human support. Such studies would help identify which strategies are most effective in reducing digital barriers and improving age-friendly access to digital health services.

## 6 Conclusion

In conclusion, this study shows that older caregivers face substantial barriers when using digital health technologies in caregiving contexts. These barriers are not limited to individual digital skills; rather, they are shaped by the interaction of age-unfriendly interface design, cognitive burden, limited trust, and insufficient family and professional support. By examining the experiences of older caregivers in China, this study highlights how such barriers may accumulate into a form of digital disability, making digital health services difficult to access, understand, and use in everyday care. Addressing this problem requires more than providing digital devices or online services. Instead, age-friendly design, practical digital skills training, and timely human assistance need to be embedded within digital health systems. A collaborative support network involving families, communities, hospitals, and government agencies may further help reduce digital exclusion. These findings suggest that digital health development should become more user-centered and age-inclusive. Reducing digital barriers for older caregivers is essential for improving equitable access to digital health services and supporting healthy aging in an increasingly digital society.

## Data Availability

The original contributions presented in the study are included in the article/supplementary material, further inquiries can be directed to the corresponding authors.
